# Activation of the DNA damage response in vivo in synucleinopathy models of Parkinson’s disease

**DOI:** 10.1038/s41419-018-0848-7

**Published:** 2018-07-26

**Authors:** Chiara Milanese, Silvia Cerri, Ayse Ulusoy, Simona V. Gornati, Audrey Plat, Sylvia Gabriels, Fabio Blandini, Donato A. Di Monte, Jan H. Hoeijmakers, Pier G. Mastroberardino

**Affiliations:** 1 Department of Molecular Genetics, Rotterdam, The Netherlands; 2 Laboratory of Functional Neurochemistry, IRCCS Mondino Foundation, 27100 Pavia, Italy; 3 German Centre for Neurodegenerative Diseases (DZNE), 53175 Bonn, Germany; 4 Department of Neuroscience, Erasmus MC, 3015 GE Rotterdam, The Netherlands; 5 Princess Máxima Center for Pediatric Oncology, Utrecht, The Netherlands; 6 CECAD, University of Cologne, Cologne, Germany

## Abstract

The involvement of DNA damage and repair in aging processes is well established. Aging is an unequivocal risk factor for chronic neurodegenerative diseases, underscoring the relevance of investigations into the role that DNA alterations may have in the pathogenesis of these diseases. Consistently, even moderate impairment of DNA repair systems facilitates the onset of pathological features typical of PD that include derangement of the dopaminergic system, mitochondrial dysfunction, and alpha-synuclein stress. The latter establishes a connection between reduced DNA repair capacity and a cardinal feature of PD, alpha-synuclein pathology. It remains to be determined, however, whether alpha-synuclein stress activates in vivo the canonical signaling cascade associated with DNA damage, which is centered on the kinase ATM and substrates such as γH2Ax and 53BP1. Addressing these issues would shed light on age-related mechanisms impinging upon PD pathogenesis and neurodegeneration in particular. We analyzed two different synucleinopathy PD mouse models based either on intranigral delivery of AAV-expressing human alpha-synuclein, or intrastriatal injection of human alpha-synuclein pre-formed fibrils. In both cases, we detected a significant increase in γH2AX and 53BP1 foci, and in phospho-ATM immunoreactivity in dopaminergic neurons, which collectively indicate DNA damage and activation of the DNA damage response. Mechanistic experiments in cell cultures indicate that activation of the DNA damage response is caused, at least in part, by pro-oxidant species because it is prevented by exogenous or endogenous antioxidants, which also rescue mitochondrial anomalies caused by proteotoxic alpha-synuclein. These in vivo and in vitro findings reveal that the cellular stress mediated by alpha-synuclein—a pathological hallmark in PD—elicits DNA damage and activates the DNA damage response. The toxic cascade leading to DNA damage involves oxidant stress and mitochondrial dysfunction The data underscore the importance of DNA quality control for preservation of neuronal integrity and protection against neurodegenerative processes.

## Introduction

Parkinson’s disease (PD) is the second most common neurodegenerative disorder after Alzheimer’s. PD is characterized by severe loss of dopaminergic neurons in the *substantia nigra pars compacta* (SNpc) and it has been traditionally considered the prototypical movement disorder. More recently, however, growing consensus indicates that PD pathology affects multiple domains and is not confined to motor function, therefore indicating that PD is rather a multisystem and complex disorder^1^.

PD pathogenesis proceeds along converging pathways that impact fundamental cellular functions such as protein homeostasis and mitochondrial function, and particularly accumulation of proteotoxic oligomeric alpha-synuclein (α-syn) and reduced activity of respiratory complex I^2,3^. Consistently, overexpression of α-synuclein, intracerebral seeding of proteotoxic α-synuclein pre-formed fibrils (PFF), and toxicological inhibition of complex I recapitulate PD in laboratory models^4–8^.

Aging is an unequivocal risk factor for several morbidities, including chronic neurodegenerative diseases and PD^9,10^. The causes of age-related deterioration are complex and, while there is large consensus that accumulation of cellular damage in time is pivotal^11^, the nature of the mechanisms of aging that specifically promotes PD onset remain unclear. Plausibly, age-related alterations favoring a diseased state encompass multiple factors that eventually synergize to sensitize the organism; among these elements, defective maintenance of genome integrity and consequent accumulation of DNA damage may be particularly relevant. DNA is, in fact, at the highest hierarchical level of biological information and, once corrupted, may impact other biomolecules to cause profound and transmissible and/or long-lasting consequences. DNA damage, moreover, may cause persistent anomalies in post-mitotic and non-replaceable cells such as neurons, unless efficient repair mechanisms amend the lesions in the double-helix. Consistently, laboratory models and patients with defects in certain DNA repair pathways, for instance in transcription-coupled and nucleotide excision repair (TCR and NER respectively), display a progeroid phenotype in which accelerated aging is paralleled by neurological defects^12^. Importantly, DNA damage impacts mechanisms that are central to PD pathogenesis, including mitochondrial function, redox homeostasis, and protein quality control^13^.

Collectively, these elements underscore the relevance of investigations into the role that DNA alterations may have in the pathogenesis of these diseases^14^. We have recently shown that even moderate impairment of DNA repair systems - including NER, transcription-coupled repair (TCR), and double-strand break repair pathways (DSB) - facilitates the onset of pathological features typical of PD that include derangement of the dopaminergic system, mitochondrial dysfunction and complex I activity suppression, and increased alpha-synuclein phosphorylation^13^. The latter establishes a connection between reduced DNA repair capacity and a cardinal feature of PD, synuclein pathology.

It remains to be determined, however, whether alpha-synuclein stress activates in vivo the canonical signaling cascade associated with DNA damage, i.e., the DNA damage response (DDR), which can be monitored by following its key players ATM, γH2AX, and 53BP1. The large kinase ATM is recruited at the site of lesions such as DSBs to phosphorylate *in cis* the histone H2AX (γH2AX)^15^. γH2AX marks the initiation of a nucleation process resulting in the formation of characteristic γH2AX nuclear *foci*, which leads to additional ATM recruitment at the damage site, thereby escalating the kinase activity^16^. The nucleation process is facilitated by mediator such as 53BP1, which also forms *foci* at the site of damage^17–19^. In light of the connection between DDR and senescence^20^, and more in general between DNA damage accumulation and aging, addressing whether α-synuclein-mediated proteotoxicity triggers the DDR may shed light on age-related, crucial mechanisms impinging upon PD pathogenesis and more in general on neurodegeneration. We therefore explored DDR activation in two different synucleinopathy in vivo models of PD and investigated possible causative mechanisms in vitro.

## Materials and Methods

### Chemicals

Sodium Nitrite (563218), oligomycin (75351), FCCP (C2920), rotenone (557368) and antimycin (A8674), N-acetyl-cysteine (NAC, A7250), were obtained from Sigma-Aldrich (St. Louis, MO, USA).

### Animal care

Animals were kept on a regular diet and housed at the Animal Resource Center (Erasmus University Medical Center), which operates in compliance with the “Animal Welfare Act” of the Dutch government, following the “Guide for the Care and Use of Laboratory Animals” as its standard.

### In vitro synthesis of α-synuclein pre-formed fibrils (PFF)

α-Synuclein pre-formed fibrils (PFF) have been generated from human α-synuclein monomer in assembly buffer (PBS, pH 7.0) according to the manufacturer’s protocol (Proteos). Briefly, 4 mg of monomeric α-syn were shacked at 1000 r.p.m. for 7 days at 37 °C. Fibrils were then aliquoted and stored at −80 °C in single use vials. The efficiency of the fibrillation reaction was verified with Thioflavin T (25 µM, Sigma, T 3516) fluorimeter.

### Stereotaxic injection of AAV particles and α-syn pre-formed fibrils (PFF)

Recombinant adeno-associated virus (serotype 2 genome and serotype 6 capsid, AAV) was used to express human h-α-syn in the mouse *substantia nigra*. Gene expression was under the control of a human Synapsin 1 promoter and enhanced using a woodchuck hepatitis virus post-transcriptional regulatory element (WPRE) and a polyA signal downstream to the α-syn sequence. AAV vector production, purification, concentration and titration were performed by Sirion (Martinsried, Germany).

C57BL/6 mice were housed under a 12-h light/12-h dark cycle with free access to food and water. Surgeries were performed under isoflurane anesthesia using a stereotaxic frame (Stoelting, Ireland). Injections was made at a rate of 0.4 µl/min using a Hamilton syringe fitted to a glass capillary. The capillary was left in position for an additional 5 min before being retracted. To induce hα-syn expression, *n* = 16 mice were injected with 1.5 µL of 8.0 × 1012 genome copies/mL AAV-α-syn into the right *substantia nigra*. Stereotaxic coordinates were 2.3 mm posterior and −1.1 mm lateral to Bregma, and 4.1 mm ventral to dura mater.

PFF injections were made into the striatum of *n* = 16 mice. 2.5 µL of sonicated PFF (5 µg) were injected under isoflurane anesthesia at the following coordinates: 0.2 mm anterior and 2.0 mm lateral to Bregma, and 2.6 mm ventral to the dura mater.

### Immunohistochemistry

Immunohistological sections were processed as previously described^21^. Briefly, free floating sections were first incubated in hydrogen peroxide (H_2_O_2_, Sigma-Aldrich, St. Louis, MO, USA) 3% in PBS for 30 min to block internal peroxidases activity, and subsequently in PBS-Triton X-100 0.2% (PBS-T) and normal horse serum (NHS) 10% for 1 h at RT. Specimens were then incubated for 24 h at 4 °C with mouse monoclonal anti-Tyrosine hydroxylase (1:4000, MAB318, Millipore, MA, USA) or with affinity purified anti human alpha-synuclein (1:3000, AB5038P, Millipore,), in PBS-T and 1.5% NHS. After several washes with PBS-T, sections were incubated with biotinylated goat anti-rabbit IgG (1:500; BA 1000, Vector Laboratories, Burlingame, CA, USA), in PBS and 1% NHS for 1 h at RT. Immuno-complexes were revealed by Vectastain Elite ABC kit (PK 4000, Vector Laboratories, Burlingame, CA, USA), using 3,3’-diamino-benzidine (DAB Substrate kit for Peroxidase, SK 4100, Vector Laboratories, Burlingame, CA, USA). Finally, sections were dehydrated and mounted with Eukitt (Kindler GmbH & Co.). Slides were observed with an Olympus BX 51(Olympus, Parkway Center Valley, PA, USA) microscope equipped with a Leica DFC 420 camera.

### Quantification of striatal TH density

Striatal lesion in DAB stained sections was calculated as the extension of the lesioned area, detected by the absence of TH staining, and expressed as percentage of TH immunopositive area measured on the contralateral side (for the AAV model) or on the PBS control mice (for the PFF model). A total of 5 sections per animals were analyzed.

### Immunofluorescence

In immunofluorescence experiments, sections were processed as described in the immunohistochemistry section with minor modifications. Floating sections were incubated overnight at 4 °C with a primary antibodies. The following antibodies were used: mouse monoclonal anti-Tyrosine Hydroxylase (1:4000, MAB318, Millipore), sheep polyclonal anti-Tyrosine Hydroxylase (1:4000; NB300-110, Novus Biologicals), anti-phospho-α-Syn (S129; 1:1000; ab59264, Millipore, Germany), anti-γH2AX (1:1000; 05336, Millipore), anti-phospho ATM (05–740, 1:1000 Upstate Biotechnology); anti 53BP1 (1:1000, 05726, Millipore); Anti- GFP (1:2000; 290-50, Abcam).

Sections were rinsed with PBS and then incubated for 2 h at RT in TBS containing 0.4 % Triton X-100, 2% NHS, Alexa488 conjugated donkey anti-rabbit IgG 1:500 (Invitrogen), Alexa594-conjugated donkey anti-mouse IgG 1:500 (Invitrogen), CY3-conjugated anti-sheep IgG (1:500, Jackson). Image acquisition was performed in a Leica TCS SP5 confocal microscope. The detection parameters were set in the control samples and were kept constant across specimens. Images were analyzed in a semi-automated fashion using the Metamorph software (Molecular Devices). The software automatically generated regions of interest (ROI) on the basis of the signal of the cellular marker (TH) and of the nuclei. The software quantified the number of foci within the regions of interest. Values are expressed as the ratio of number of cells presenting more than 5 foci over the total dopaminergic neurons that have been analyzed. At least 50 cells per case were analyzed.

### Unbiased stereological counts

Unbiased stereological estimation of the total number of the dopaminergic cells in SNpc was made using the optical fractionator method^22^ from the STEREO INVESTIGATOR program on a Neurolucida computer-controlled microscopy system (Microbrightfield Inc., Williston, VT, USA). The edges of the SNc in the rostro-caudal axis were defined at all levels, with reference to a coronal atlas of the mouse brain^23^. TH-positive cells in the SNc of both hemispheres were counted in every three section, on comparable sections for all the subgroups of treatment throughout the entire nucleus. Counting frames (60 × 60 µm) were placed at the intersections of a grid (frame size 120 × 120 µm) that was randomly placed over the section. For counting, only those counting frames in which at least a part of the frame fell within the contour of the SNpc were used. Only in focus, TH-positive cell bodies were marked. Guard volumes (3 µm from the top and 3 µm from the bottom of the section) were excluded from both surfaces to avoid the problem of lost caps. The reliability of the estimate was assessed by calculation of the coefficient of as previously described^24^.

### Cell culture and immunocytochemistry

SH-SY5Y neuroblastoma cells were maintained in DMEM medium, 10% FCS and 1% pen/strep. For differentiation, cells were seeded at a cell density of 50 × 10^4^ on coverslip coated with poly-Ornithine (P4957, Sigma) and laminin (L4544, Sigma) and have been differentiated for 8 days in DMEM 1% FCS and retinoic acid (10 µM, R2625 Sigma).

Differentiated neurons were incubated with PFF (1 µg/mL) for 18 h and processed for immunocytochemistry. Briefly, cells were fixed in PFA 4% for 20′ and, after washes in PBS, were blocked in 3% BSA, 0,1% Triton X-100 and PBS for additional 20'. Cells were then incubated with an anti-synuclein antibody (1:2000; Ab6162, Abcam), an anti-γH2AX (1:1000; 05336, Millipore), or an anti 53BP1 (1:1000, 05726 Millipore) for 3 h at room temperature (RT). After 3 washes with PBS, cells were incubated for 1 h with the following secondary antibodies: Alexa488 conjugated donkey anti-rabbit IgG 1:1000 (Invitrogen), Alexa594-conjugated donkey anti-mouse IgG 1:1000 (Invitrogen), Cy3-conjugated anti- sheep IgG (1:1000, Jackson). Image acquisition was performed using a Zeiss SP5 confocal microscope. The detection parameters were set in the control samples and were kept constant across specimens. Images were analyzed in a semi-automated manner using the Metamorph software (Molecular Devices). The software automatically generated regions of interest (ROI) on the basis of the signal of the nuclei. The software quantitated the number of foci within the regions of interest and values are expressed of number of cells presenting more than 5 foci over the total dopaminergic neurons that have been analyzed; a minimum amount of 50 cells per case have been counted.

### Bioenergetics profiling

The Seahorse XF24 Extracellular Flux Analyzer (Agilent Technologies, Santa Clara, Ca, USA) was used to generate the bioenergetics profiles as previously described^25^. SH-SY5Y cells were seeded at a density of 6 × 10^4^ cells per well and allowed to adhere overnight. After adhesion, cells were treated with NAC (1 mM), PFF (10 µg/mL), or vehicle (PBS) dissolved/diluted in growth medium for 18 h or pre-treated for 48 h with sodium nitrite (10 µM) or vehicle (PBS) before PFF exposure for 18 h. Media were refreshed daily. On the experimental day, cells medium was changed to unbuffered DMEM (XF Assay Medium – Agilent Technologies, Santa Clara, Ca, USA) supplemented with 5 mM glucose and 1 mM sodium pyruvate, and incubated 1 h at 37˚ C in absence of CO_2_. Medium and reagents were adjusted to pH 7.4 on the day of the assay. After four baseline measurements for the oxygen consumption ratio (OCR), cells were sequentially challenged with injections of mitochondrial toxins: 0.5 µM oligomycin (ATP synthase inhibitor), 1 µM FCCP (mitochondrial respiration uncoupler), 0.5 µM rotenone (complex I inhibitor), and 0.5 µM antimycin (complex III inhibitor).

### Statistical analysis

All values are expressed as mean ± s.e.m. Statistical significance was assessed by two sided Student’s *t* test or one-way analysis of variance (ANOVA) followed by the Dunnet’s multiple comparison post hoc test. In all instances a value of *P* < 0.05 was considered as a threshold for statistical significance.

## Results

### Proteotoxic α-syn activates the DNA-damage response

**Fig. 1:**
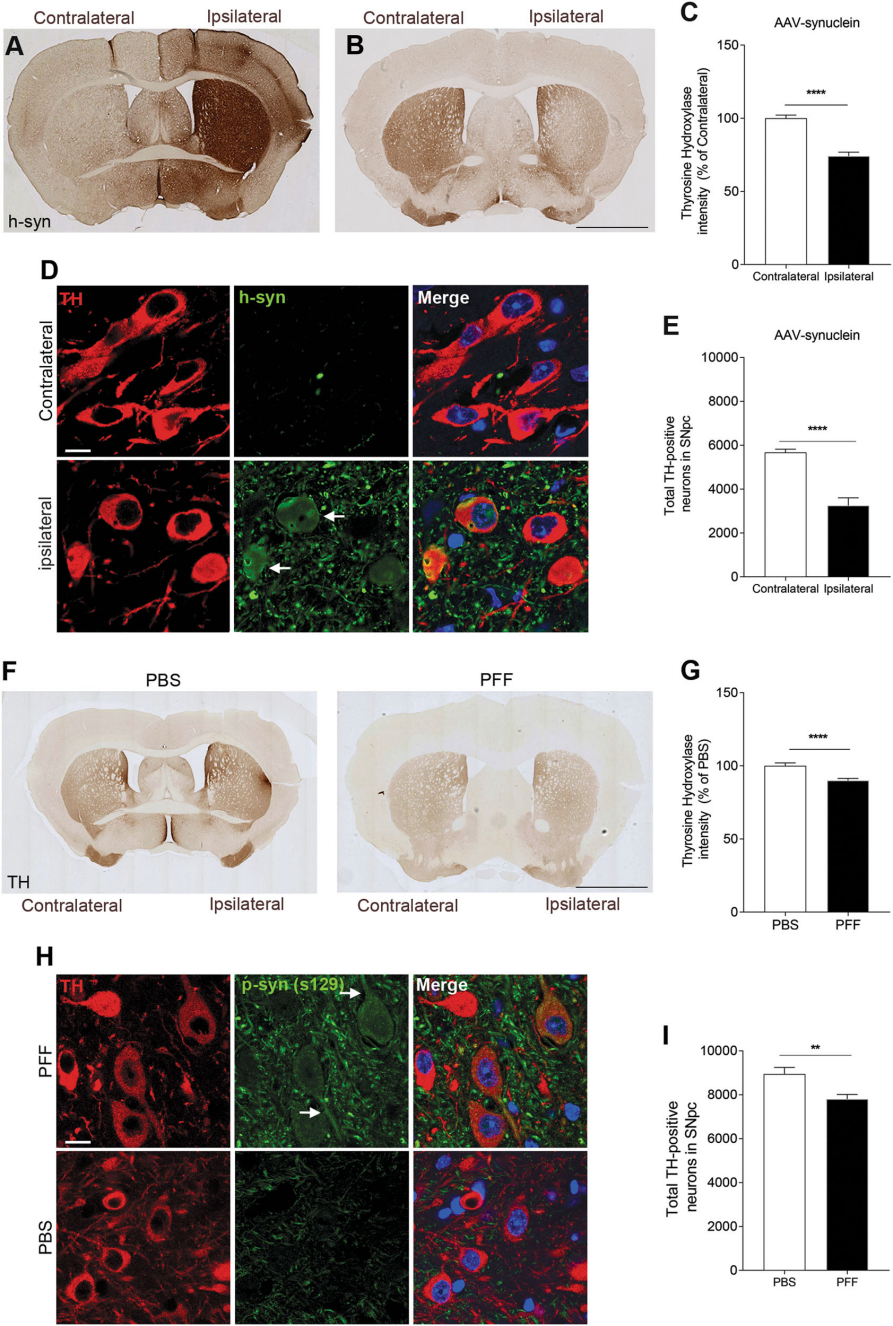
α-Synuclein  overexpression or intracerebral seeding impacts the dopaminergic system. **a**–**c** Intranigral injection of AAV2/6 serotype expressing human α-syn (h-syn) results in increased protein expression paralleled by reduction in tyrosine hydroxylase (TH) levels. (**d**) h-syn is also expressed in dopaminergic cell bodies (arrows). **e** Unbiased stereological counts demonstrate a reduction in nigral dopaminergic cell bodies. **f**, **g** Intracranial injection of α-syn pre-formed fibrils (PFF) causes striatal dopaminergic denervation as evidenced by a reduction in TH immunoreactivity. **h** Increased levels of ser129-phosphosynuclein in the substantia nigra (arrows) indicate α-syn stress in PFF injected animals. **i** Unbiased stereological counts showing a decrease in dopaminergic cell bodies in the substantia nigra. Scale bars: 1 mm in **a**, **b** 50 μm in **d**. (**p < 0.01; ***p < 0.001; Student’s *t* test). All bar graphs show mean + /− s.e.m

To study DDR activation induced by proteotoxic stress in vivo, we took advantage of two different synucleinopathy mouse models of PD based either on intranigral delivery of AAV-expressing human α-syn (AAV-syn)^26^, or on intrastriatal injection of human α-syn pre-formed fibrils (PFF)^8^. Six months after stereotaxic injection, AAV-syn transduced mice displayed significant striatal increase of human α-syn protein levels in the ipsilateral hemisphere, which was paralleled by a remarkable reduction of striatal tyrosine hydroxylase (TH) immunoreactivity (Fig. 1a–c). Increased α-syn was also detected inside dopaminergic cell bodies in the SNpc (Fig. 1d). As expected, these alterations were associated with significant ipsilateral reduction in nigral dopaminergic cell bodies, as detected by unbiased stereological counts (Fig.1e).

Lesion in the dopaminergic system was detected also in PFF treated mice, 4 months after injection, which showed reduced striatal TH immunoreactivity (Fig. 1f,g) paralleled by α-synuclein stress—as evidenced by increased phospho-synuclein levels (p129-syn) (Fig. 1h)—and reduced dopaminergic cell bodies in the SNpc (Fig. 1i), when compared to PBS injected animals. Collectively, and consistently with the original reports^5–8^, these elements indicate successful modeling of dopaminergic pathology in animals transduced with AAV-syn or exposed to proteotoxic α-syn.

**Fig. 2:**
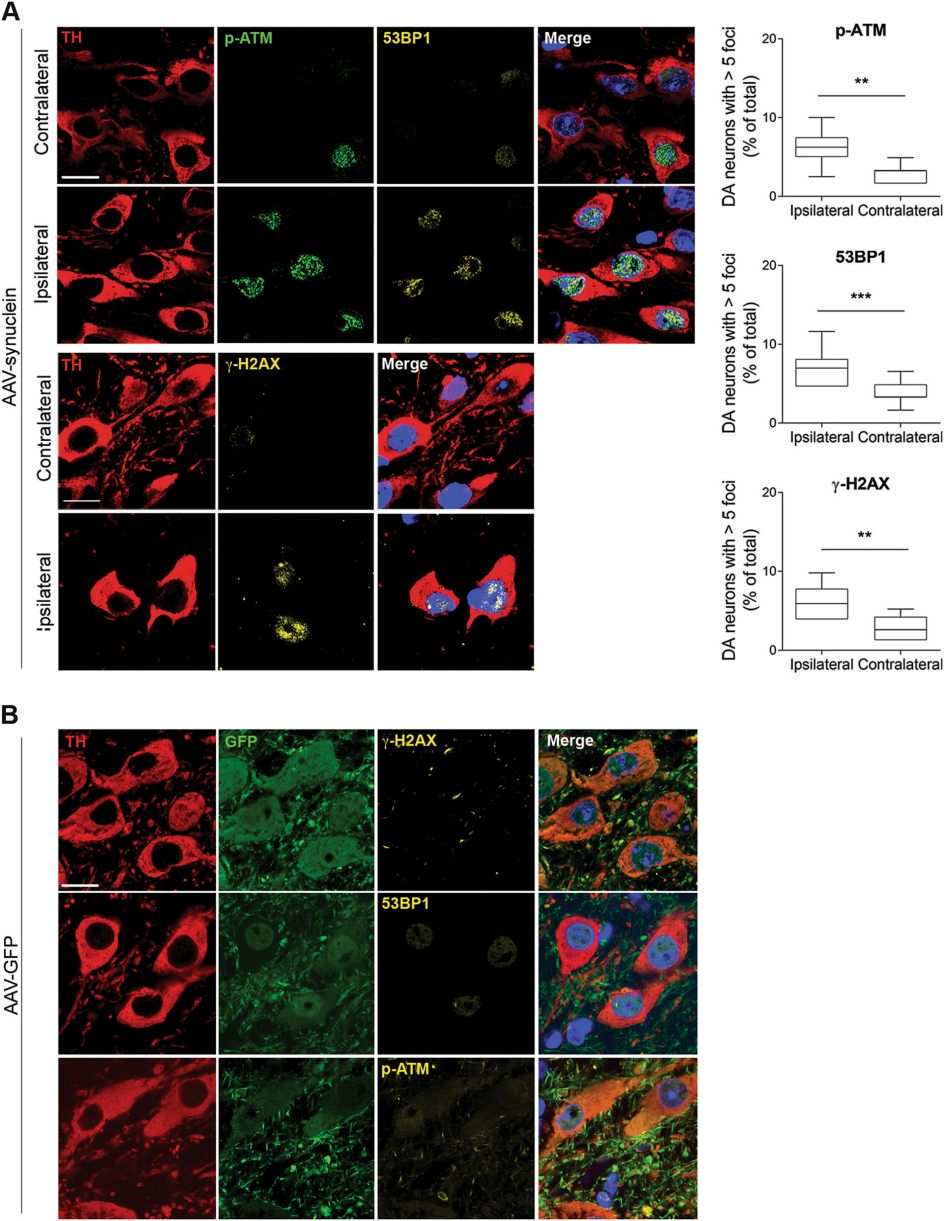
Activation of the DDR in mice transduced with AAV2/6 h-syn. **a** h-syn expression increases 53BP1 and γH2AX foci, and ATM phosphorylation in nigral dopaminergic neurons. **b** The DDR is not activated by viral delivery of GFP. Scale bar: 50 μm. (**p < 0.01; ***p < 0.001; Student’s *t* test). All bar graphs show mean + /− s.e.m

When we examined DDR activation in AAV-syn mice, we detected a significant ipsilateral increase in the DDR markers γH2AX and 53BP1 *foci*, and phospho-ATM immunoreactivity (Fig. 2a). To rule out the possibility that DDR activation under these experimental conditions may be a mere consequence of non-specific viral toxicity and/or non-specific protein overexpression, a separate group of animals was injected intrastriatally with AAVs carrying green fluorescent protein (GFP) rather than human α-syn DNA. Results showed lack of significant changes within AAV-GFP-transduced neurons, supporting the conclusion that DDR activation is indeed a specific toxic effect caused by increased α-syn expression within nigral dopaminergic cells (Fig. 2b).

**Fig. 3:**
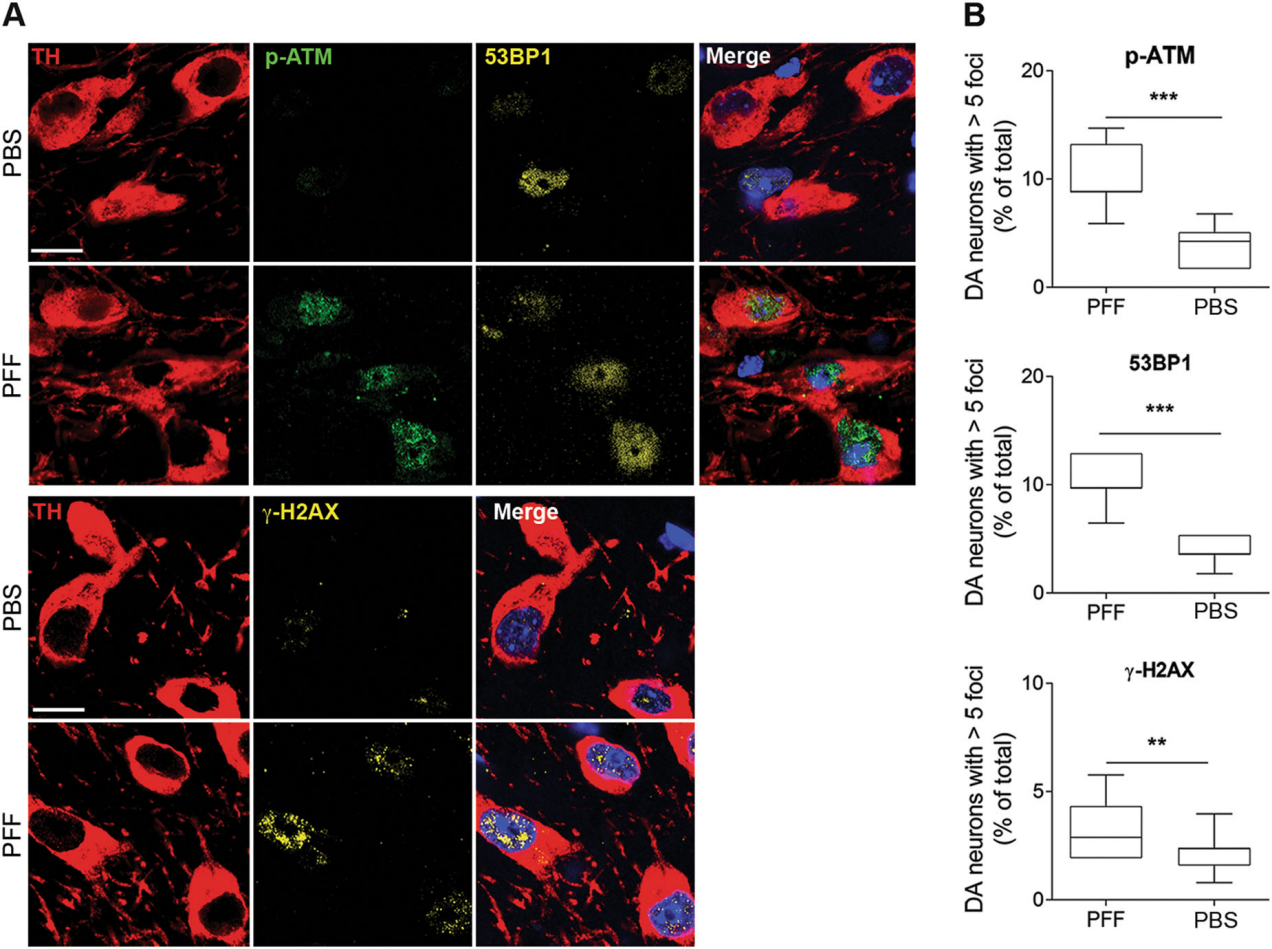
Activation of the DDR in mice injected with α-syn PFF in the striatum. Augmented levels 53BP1 and γH2AX foci, and ATM phosphorylation in dopamine neurons of the substantia nigra. Scale bar: 50 μm. (**p < 0.01; ***p < 0.001; Student’s *t* test). All bar graphs show mean + /− s.e.m

DDR hallmarks were detectable also in nigral dopaminergic neurons of PFF, but not in vehicle-injected mice (Fig.3a, b). Altogether, these elements demonstrate a causative link between α-syn proteotoxicity and DNA damage in dopaminergic neurons.

### Oxidation contributes to DDR activation

**Fig. 4:**
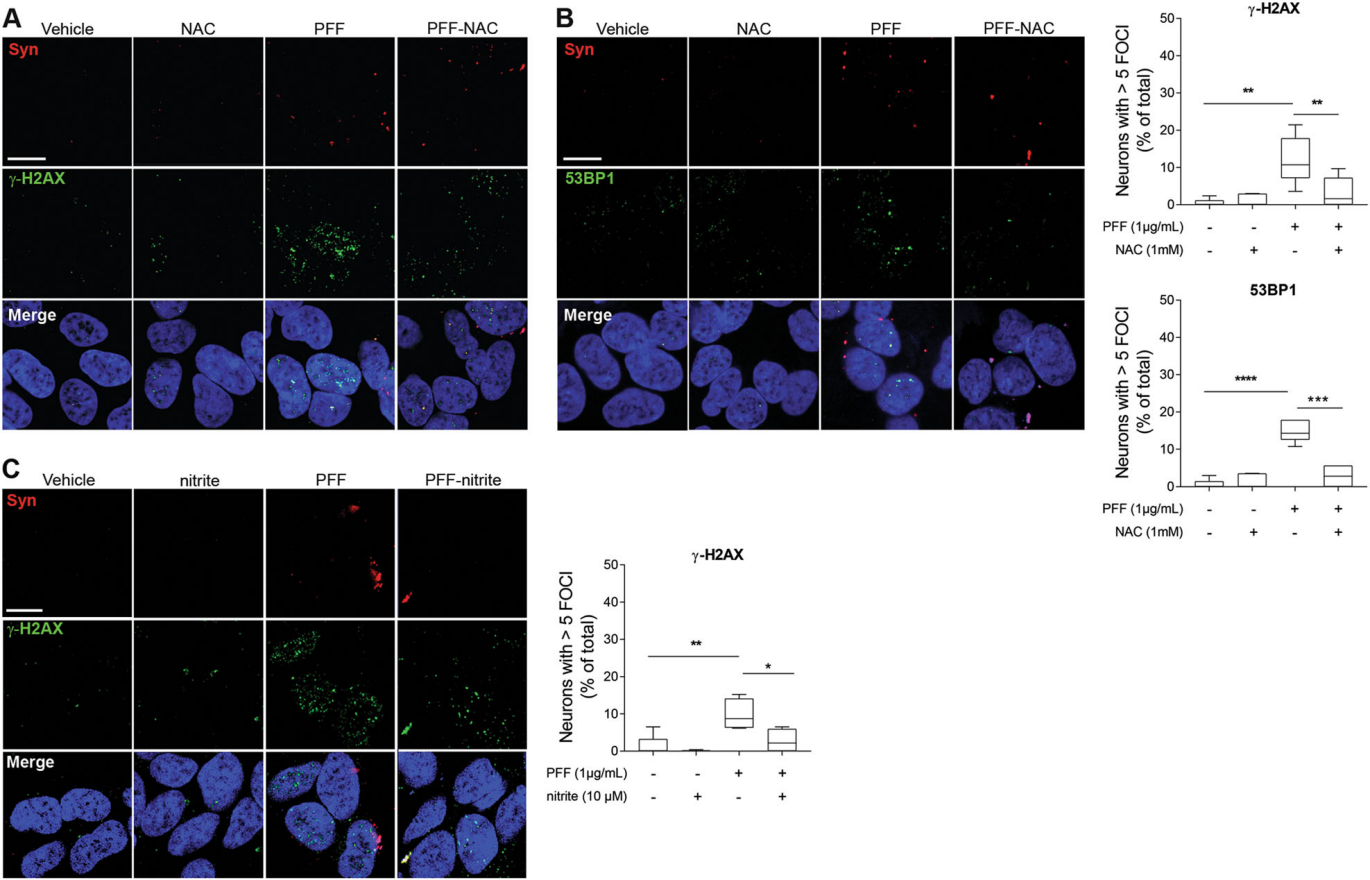
Pro-oxidant species contribute to α-syn-mediated activation of the DDR. **a**, **b** Administration of the antioxidant NAC mitigates formation of PFF-mediated γH2AX and 53BP1 foci in differentiated SH-SY5Y cells. **c** Administration of the nitric oxide donor nitrite, which activates the endogenous antioxidant Nrf2 pathway, reduces formation of PFF-mediated γH2AX foci. Scale bars: 50 μm. (*p < 0.05; **p < 0.01; ***p < 0.005; ****p < 0.001; one-way ANOVA followed by Dunnet’s multiple comparison post doc test.) All bar graphs show mean + /- s.e.m

To explore the intracellular mechanisms responsible for DDR activation mediated by proteotoxic α-syn species, we took advantage of the dopaminergic cell line SH-SY5Y, which has been used by others and ourselves to explore selected PD mechanisms in vitro^25,27^. Given that pro-oxidant reactive species (RS) are associated with α-syn proteotoxicity^28^, have been involved in PD pathogenic mechanisms^29^, and are major genotoxic agents^30^, we hypothesized that DDR activation might be caused, at least in part, by excessive oxidation. We therefore evaluated the effects of the thiol-based RS scavenger and antioxidant N-acetylcysteine (NAC) on DDR activation in differentiated SH-SY5Y cells exposed to PFF. Indeed, NAC reduced levels in both γ-H2AX and 53BP1 *foci* (Fig. 4a, b).

To substantiate these findings, we used a parallel strategy relying on endogenous rather than exogenous antioxidants. We therefore treated cells with the nitric oxide (NO) donor nitrite, which we have shown to trigger expression of endogenous antioxidant genes via S-nitrosation of Keap1 and consequent activation of the Nrf2 pathway^25^. We found that administration of nitrite significantly reduced PFF-induced formation of γ-H2AX *foci* (Fig. 4c). Collectively, these elements indicate that pro-oxidants mechanistically participate in DDR activation induced by proteotoxic α-syn.

### DDR-activating oxidation impairs mitochondrial function

**Fig. 5:**
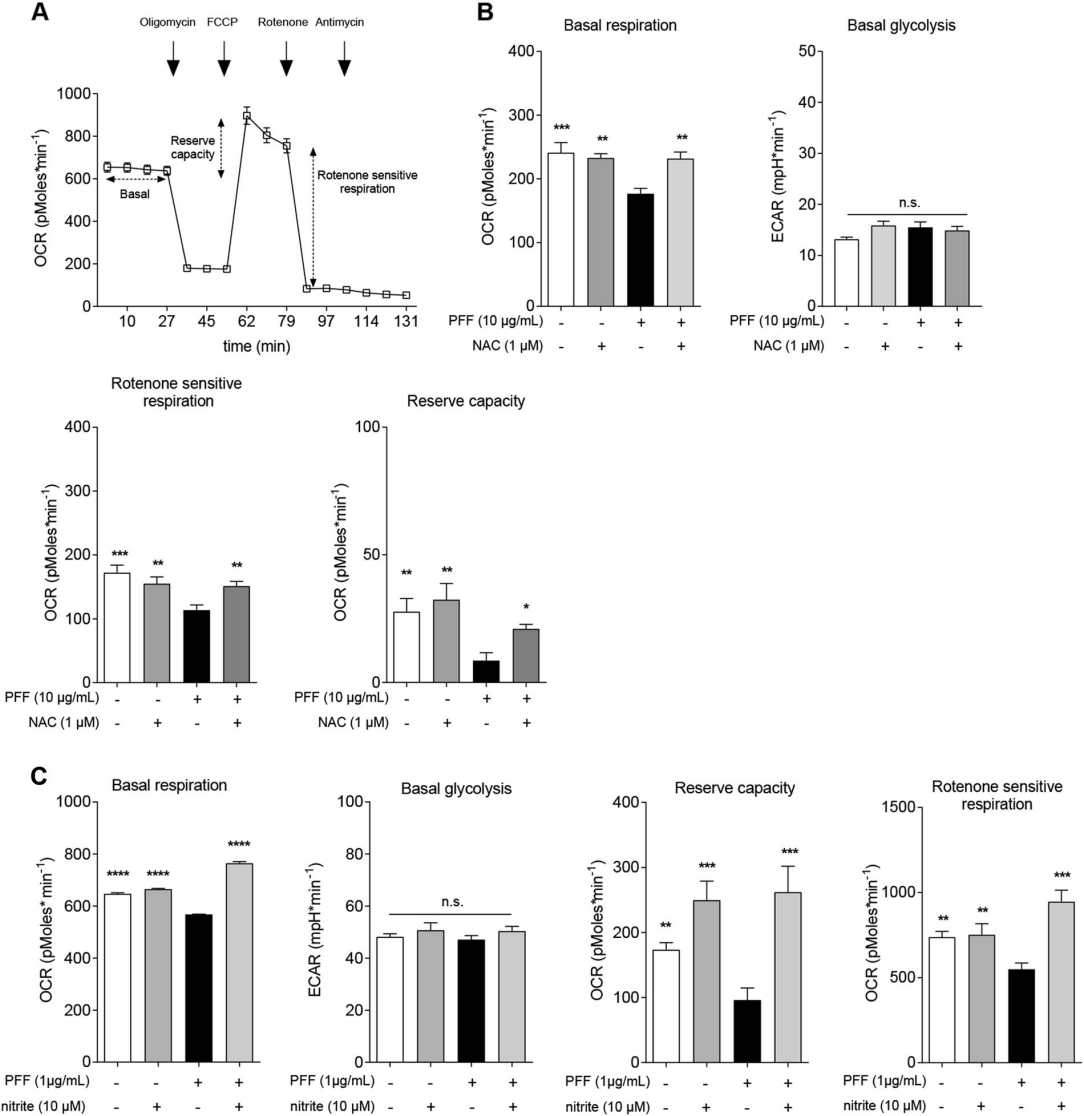
Potentiation of exogenous or endogenous antioxidants reverses mitochondrial anomalies induced by PFF in SH-SY5Y cells. **a** Representative trace of a microscale oxygraphy experiment. **b** NAC or **c** nitrite treatments restore basal and rotenone sensitive respiration, as well as reserve capacity in SH-SY5Y cells exposed to PFF. (*p < 0.05; **p < 0.01; ***p < 0.005; ****p < 0.001; one-way ANOVA followed by Dunnet’s multiple comparison post doc test.) Graphs represent mean ± s.e.m

Mitochondria are the major intracellular source of RS and when damaged - for instance at the level of the electron transport chain - production of pro-oxidants such as superoxide may increase^31^. Proteotoxic α-syn may directly cause mitochondrial dysfunction via physical interaction with these organelles^32^. Moreover, defective DNA repair is sufficient to impact mitochondrial function in the ventral mesencephalic brain region containing the SNpc^13^. On these premises, and given the prominent role of mitochondrial anomalies in PD^29^, we explored whether DDR-activating oxidation caused by α-syn toxicity might impact on mitochondrial function as well. We therefore evaluated mitochondrial bioenergetics in SH-SY5Y cells exposed to PFF and found that proteotoxic stress reduced both basal respiration, reserve capacity, and rotenone sensitive respiration, i.e., attributable to complex I (Fig. 5a). Potentiation of exogenous or endogenous antioxidant defenses by NAC or nitrite supplementation, which attenuated the DDR, also reversed these defects (Fig. 5a, b). This last set of experiments therefore substantiated the nexus between proteotoxicity, oxidative stress, DNA damage, and mitochondria.

## Discussion

α-syn containing Lewy bodies are a neuropathological hallmark of PD, their distribution correlates with clinical symptoms^33^, and aggregated α-synuclein causes dopaminergic cell death in laboratory animal models^8^. The major risk factor for PD is aging, which has been associated to the accumulation of macromolecular damage, particularly in DNA. In fact, accumulation of DNA damage secondary to defective repair systems causes accelerated aging in patients and in laboratory animals^13^. Effective genome maintenance is essential for proper brain function, as indicated by neurological defects - also at the dopaminergic system level - in organisms with defective repair mechanisms^34,35^. On these premises, genomic instability is considered a hallmark of aging^11^ and DNA damage has been recently hypothesized to constitute a unifying element across different neurodegenerative disorders^14^. It is therefore conceivable that the interplay between a major pathologic feature of PD and a crucial attribute of aging—i.e., α-synuclein toxicity and DNA damage—may constitute an important mechanism linking PD to its main risk factor. However, whether α-synuclein could cause DNA damage in vivo had not been investigated thus far.

Here we used two different rodent models to show that synucleinopathy causes upregulation of three different markers of DNA damage in vivo—γH2AX, 53BP1, and p-ATM—specifically in dopaminergic neurons. Mechanistic experiments in cell cultures show that this effect depends, at least in part, on ROS production because it is reduced by the enhancement of exogenous or endogenous antioxidant defenses. Our in vivo results are consistent with previous in vitro evidence showing that α-synuclein can cause DNA breaks and that the effect is exacerbated by its oligomerization or oxidation^36^. It should be emphasized, however, that H2AX phosphorylation is not uniquely associated to DSB and can also occur upon formation of other types of lesions, for instance single strand breaks^37^. This evidence, together with our previous data demonstrating synucleinopathy in mice with mild NER defect^13^, suggests that α-synuclein triggers a broad range of DNA lesions, rather than a single, specific type of damage. Moreover, while our results are specific for α-syn, it is plausible that also other proteotoxic species—e.g., amyloid-beta—might activate DDR. Other studies to evaluate this possibility are warranted.

**Fig. 6:**
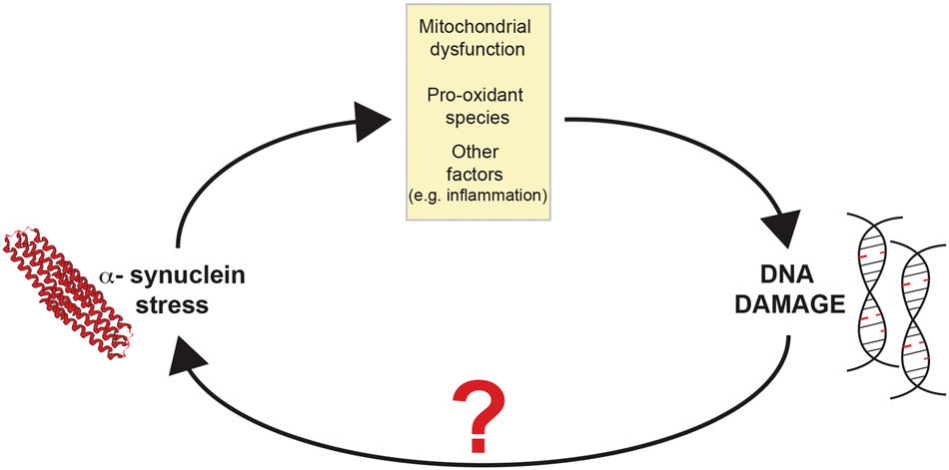
Schematic illustrating the proposed model in which proteotoxic stress and DNA damage accumulation synergize to trigger an escalating positive feedback loop. α-Synuclein proteotoxicity impacts genomic integrity, which in turn promotes α-synuclein stress. α-synuclein-mediated DNA damage involves, but is not limited to the participation of mitochondrial pro-oxidant species, and other factors may contribute to α-synuclein-mediated DNA damage. The mechanisms that cause α-synuclein stress following DNA damage are unknown.

DNA damage may be particularly noxious in post-mitotic cells; genomic integrity might be particularly threatened in DA neurons, given their intrinsically oxidized intracellular redox state even in normal physiological conditions^21,38^. Accordingly, several studies at single-cell level reported increased levels of deletions in dopaminergic neurons’ mtDNA in PD^39,40^. Additional evidence indicates that mtDNA may be particularly sensitive to damage in DA neurons^41^. While collectively these elements lend support to a pathogenic role for DNA damage in PD, nuclear genomic integrity in dopaminergic neurons—in both the diseased and the healthy aging brain—has not received equal attention, despite the vast majority of mitochondrial proteins are encoded by nuclear DNA. This aspect is particularly relevant in light of recent findings from our laboratory showing reduced DNA repair capacity in peripheral fibroblasts of PD patients, which in PD could synergize with high oxidation to activate the pathogenic cascade^13^. More in general, mild genomic instability caused by defective NER impacts on the dopaminergic system, causing alterations that parallel those observed in PD, and is sufficient to cause increased oxidation as well as α-synuclein stress, as evidenced by increased phosphorylated-synuclein levels in DA neurons. Besides indicating that aging-related genomic instability predisposes to synucleinopathy, this evidence demonstrated that DNA damage is per se sufficient to trigger proteotoxicity and is also consistent with other findings showing that DNA damage triggers ROS generation by the NADPH oxidase system via H2AX phosphorylation^42^. An escalating feedback loop mechanism might be therefore envisioned, in which accumulation of DNA damage caused by aging and/or defective repair systems promote α-syn stress—also via abnormal oxidation—which in turn causes further DNA damage (Fig. 6).

In cell cultures exposed to proteotoxic α-synuclein, we showed that DDR activation is paralleled by perturbation of mitochondrial function and that both anomalies are mitigated by potentiation of exogenous or endogenous antioxidants defenses. This evidence establishes a connection between α-synuclein stress, DDR, oxidation, and the major intracellular site of RS production, i.e., mitochondria, and is consistent with the data showing that α-syn physically interacts with mitochondria and that such association culminates in redox alterations^32^. Mitochondria, however, may not be the sole responsible for DNA-damaging ROS generation; inflammation, which occurs in α-syn models as well as in PD^43,44^, and activation of microglial NADPH oxidase, in particular, are also likely contributors to DNA damage. Additional sources of DNA damage may also oxidized lipids such 4-HNE, which induces the formation of toxic exocyclic adducts^45^. Moreover, DNA damage in vivo may be caused by other reactants than pro-oxidants, for instance the methyl donor metabolite S-Adenosymethionine, which alkylates DNA^46^.

Finally, our work is in line with very recent findings showing that ATM activation plays an important role in preventing protein aggregation^47^. It is therefore plausible that DDR activation following α-synuclein stress may be part of a broader biological cellular reaction to contrast aggregation.

Collectively, these data underscore the importance of DNA quality control for preservation of neuronal integrity and protection against neurodegenerative processes. Age-related impairments of DNA repair capability could predispose to toxic/pathological processes associated with the accumulation and aggregation of specific proteins - for instance α-synuclein in PD. A progressive decline of DNA quality control during aging could therefore be a crucial mechanism underlying the enhanced neuronal vulnerability of the older brain and its predisposition to neurodegenerative diseases.
